# Stem Cells in Myelodysplastic Syndromes and Acute Myeloid Leukemia: First Cousins or Unrelated Entities?

**DOI:** 10.3389/fonc.2021.730899

**Published:** 2021-08-20

**Authors:** Romane Joudinaud, Thomas Boyer

**Affiliations:** ^1^Laboratory of Hematology, Lille Hospital, Lille, France; ^2^Laboratory of Hematology, University of Amiens, Amiens Hospital, Amiens, France

**Keywords:** myelodysplastic syndromes, acute myeloid leukemia, leukemic stem cell, whole genome sequencing, single cell

## Abstract

Myelodysplastic syndromes (MDSs) are associated with a significant risk of transformation to acute myeloid leukemia (AML), supported by alterations affecting malignant stem cells. This review focuses on the metabolic, phenotypic and genetic characteristics underlying this dynamic evolution, from myelodysplastic stem cells (MDS-SCs) to leukemic stem cells (LSCs). MDS-SCs are more likely to be derived from healthy hematopoietic stem cells (HSCs), whereas LSCs may originate from healthy progenitors, mostly LMPP (lymphoid-primed multipotential progenitors). Moreover, overexpression of CD123 and CLL1 markers by LSCs and MDS-SCs in high risk-MDS [HR-MDS] has led to exciting therapeutic applications. Single-cell sequencing has suggested that clonal evolution in the stem cell compartment was non-linear during MDS initiation and progression to AML, with pre-MDS-SC acquiring distinct additional mutations in parallel, that drive either MDS blast production or AML transformation. In AML and HR-MDS, common metabolic alterations have been identified in malignant stem cells, including activation of the protein machinery and dependence on oxidative phosphorylation. Targeting these metabolic abnormalities could prevent HR-MDS from progressing to AML. Strikingly, in low risk-MDS-SC, the expression of ribosomal proteins is decreased, which may be accompanied by a reduction in protein synthesis.

## Introduction

Over 70% of adults under 60 years of age diagnosed with acute myeloid leukemia (AML) achieve complete hematologic remission following induction therapy. Nevertheless, a significant proportion of these patients experiences relapse, and the 5 year overall survival does not exceed 50% (1). At diagnosis, a higher percentage of leukemic stem cells (LSCs), also called leukemia-initiating cells, confers an increased risk of relapse and resistance to treatment, as well as a lower overall survival (2, 3). Similarly, persistence of LSCs during a complete hematologic remission is associated with poorer overall survival (2).

Myelodysplastic syndromes (MDSs) are associated with a significant risk of transformation to AML, the 5-year transformation risk amounting to 20% (4). Improved phenotypic, genetic and metabolic characterization of myelodysplastic stem cells (MDS-SCs), also called MDS-initiating cells, should have a strong prognostic and therapeutic impact. In this review, we highlight the recent advances in the knowledge of MDS-SCs and LSCs. A better insight into their characteristics should allow an earlier detection of AML transformations and the development of drugs directed against specific targets.

## Part 1: Phenotypic Characteristics of Malignant Stem Cells: Cell of Origin and Aberrant Markers

### MDS Is an Hematopoietic Stem Cell (HSC) Disease

Stem cell potential of hematopoietic cells can be demonstrated *in vitro* by performing cell culture on a stromal feeder for 6 weeks and ultimately evaluating the ability to give rise to hematopoietic cells, a technique referred as LTC-IC (long-term culture-initiating cells assays). However, *in vivo* approaches in murine xenotransplantation models remain the gold standard as stem cells possess the ability to regenerate long-term human multipotent hematopoiesis in serial xenografts. These *in vivo* experiments have first demonstrated that HSCs reside in the CD34+ CD38- (5), later CD34+ CD38- CD90+ (6), and finally CD34+ CD38- CD90+ CD45RA- (7) cell fraction with a weak expression of CD45 in the bone marrow.

MDS-SCs were first characterized in MDS with isolated del(5q). In this population, LTC-IC assays demonstrated that malignant stem cells were located in the CD34+ CD38-/low bone marrow fraction (8). Later, their selective chemoresistance to lenalidomide allowed to locate them in the CD34+ CD38-/low CD90+ fraction (9). Finally, the first successfully performed murine xenografts specified their CD34+ CD38- CD90+ CD45RA- phenotype, whether derived from patients with low-risk MDS (LR-MDS) [isolated del(5q)] (10) or high-risk MDS (HR-MDS) (monosomy 7) (11). In addition, CD34+ CD38+ progenitors (common myeloid progenitors [CMP], granulocyte-macrophage progenitors [GMP], and megakaryocyte-erythroid progenitor [MEP]) as well as CD34- cells failed to generate the pathology when injected in immunocompromised mice (10).

Thus, MDS-SCs display the same phenotype as physiological HSCs. The cell of origin could therefore be a normal HSC, transformed into MDS-SCs with acquisition of genetic abnormalities. The original cell could also be a normal progenitor, with HSCs-defining flow cytometry phenotype acquired upon transformation. To determine the origin of MDS-SCs, several teams have studied their gene expression profile. Gene expression profiles of MDS-SCs from MDS patients with isolated del(5q) clustered extensively with healthy HSCs, suggesting that MDS-SCs are indeed originally derived from physiological HSCs (10, 12).

### AML Is a Progenitor Disease

Among CD34+ fraction, normal progenitors can be distinguished by their differential expression of CD90, CD38, CD45RA, CD110 and CD123 antigens. Among CD38 negative cells, multipotential progenitors (MPPs, CD45RA- CD90-) (7) and lymphoid-primed multipotential progenitors (LMPPs, CD45RA+ CD90-) coexist (13). Among the more committed CD38+ progenitors, CMPs (Lin- CD123+ CD45RA-), MEPs (Lin- CD123- CD45RA-) and GMPs (Lin- CD123+ CD45RA+) can be found (14).

Xenotransplantion experiments have revealed that LSCs were enriched in the CD34+ CD38- medullary compartment of AML patients (15). Goardon et al. demonstrated that Lin- CD34+ CD38- CD90- CD45RA+ (LMPP-like) LSCs coexist with CD34+ CD38+ CD123+/low CD110- CD45RA+ (GMP-like) LSCs in 87% of AML cases. However, the LMPP-like population managed to give rise to the GMP-like population *in vitro* and *in vivo*, and not the converse, suggesting its higher stemness potential. Besides, LMPP-like LSCs expression profiles are enriched for genes upregulated in more immature AMLs, consistent with CD38- CD45RA+ LSCs being more immature than GMP-like LSCs (13). In the remaining 13% of AML cases, Lin- CD34+ CD38- CD90- CD45RA- (MPP-like) LSCs coexisted with CD34+ CD38+ CD123+ CD110- CD45RA- (CMP-like) LSCs. Another team confirmed that CD45RA is a powerful LSC marker, allowing for their detection in 65% of AML cases (16). Injection of murine GMPs transformed with the fusion protein KMT2A-AF9 gave rise to AML in immunocompromised mice, demonstrating that AML LSCs may derive from progenitor cells (17). Comparing the gene expression profiles of LMPP-like and GMP-like LSCs with those of HSCs and progenitors from healthy patients, LSCs had a gene expression profile closer to healthy progenitors corresponding to their phenotypic counterpart (LMPP or GMP) than to healthy HSCs. Moreover, LSCs overexpressed genes involved in self-renewal processes compared to their healthy counterparts (13, 17). 

MDS-SCs thus likely originate from HSCs, while LSCs may derive from progenitors. Surprisingly, an expansion of the CMP compartment has been established in LR-MDSs, mirrored by an expansion of the GMP compartment in HR-MDSs (18), these compartments being enriched with LSCs in AML (13).

### Stem Cell Malignancy Markers and Antigenic Targeting

We chose to focus on a few promising markers that have been reported in recent publications.

Flow cytometry has revealed that the CD123 antigen (interleukin-3 receptor alpha chain) was over-expressed in the LSC-enriched fraction (CD34+ CD38-) of bone marrow collected from patients diagnosed with primary AML compared to healthy patients. Purified CD34+ CD123+ leukemia cells successfully established and maintained leukemic populations after transplantation into immunocompromised mice, then demonstrating their stemness potential (19). Another team revealed that CD123 was expressed by a majority of AML blasts, with a similar mean fluorescence intensity (MFI) level compared to LSCs (20). In MDS, CD123 expression profile is stage-dependent, being overexpressed in the CD34+ CD38- bone marrow fraction of HR-MDS patients compared to LR-MDS patients (21). Targeted therapies against CD123 are therefore quickly arising (22). Since CD123 is expressed by the majority of CD34+ hematopoietic progenitors and a portion of HSCs (23, 24), toxicity of these new therapies may be limiting. Thus, identification of more specific targets is needed.

Human C-type lectin-like molecule-1 (CLL1) is expressed by malignant cells collected from the CD34+ CD38- medullar compartment of AML patients (25). The injection of CD34+ CLL1+ bone marrow fraction derived from these patients is able to generate AML in immunocompromised mice, demonstrating that CLL1 may be a relevant marker of LSC. CLL1 is also expressed on AML blasts (26). Interestingly, its expression seems stable when AML relapses (25). In MDS, CLL1 expression profile is also stage-dependent. Recently, overexpression of CLL1 by CD34+ CD38- bone marrow fraction and its various subcompartments (HSC, MPP and LMPP) has been demonstrated in patients with MDS-EB (myelodysplastic syndromes with excess blasts), compared to LR-MDS and healthy individuals (27). Although expressed by a fraction of bone marrow progenitors (26), CLL1 is not expressed in the CD34+ CD38- fraction of normal and regenerating bone marrows (25). MCLA-117, a bispecific antibody targeting both CD3 and CLL1 has demonstrated its efficacy *in vitro* and is currently being tested in a clinical trial (28) (NCT03038230). CAR-T cells targeting CLL1 have also been optimized (29, 30) and are evaluated in several clinical trials (31).

Phenotypic characteristics of malignant stem cells and healthy counterparts are summarized in **Table 1**. These markers are heterogeneously expressed by stem cell populations. Nevertheless, in AML, CD45RA may be the most interesting LSC marker as its combination with CD34, CD38 and CD90 allows to estimate these cells at diagnosis and follow up by multiparametric flow cytometry (16, 32).

**Table 1:** Phenotypic characteristics of malignant stem cells and healthy progenitors.

	CD34	CD38	CD90	CD45RA	CD123	CLL1
*LSC*	+	–	–	+	+	+
*MDS SC from LR MDS*	+	–	+	–	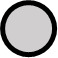	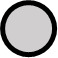
*MDS SC from HR MDS*	+	–	+	–	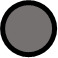	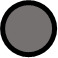
*HSC*	+	–	+	–	+/-	–
*LMPP*	+	–	–	+	low	–

For malignant (MDS-SC, LSC) and physiological (HSC, LMPP) cell populations, expression of the following antigens is depicted: CD34, CD38, CD90, CD45RA, CD123 and CLL1. The “-” sign indicates absence of expression of the marker and the “+” sign indicates frank expression of the marker. For a given marker, if the intensity of expression in a population is defined in respect to another population, the staining intensity of the circles reflect the intensity of expression. MDS-SC, myelodysplastic stem cells; LSC, leukemic stem cells; HSC, hematopoietic stem cells; LMPP, lymphoid-primed multipotential progenitors; MDS, Myelodysplastic syndromes; LR, Low risk; HR, High risk.

## Part 2: Genetic Characteristics of Malignant Stem Cells

### Genetic Defects in MDS and AML

Cytogenetic abnormalities observed in primary AML are mostly balanced, such as t(8;21)(q22;q22), t(15;17)(q22;q21), inv(16)(p13;q22)/t(16;16)(p13;q22), and translocations involving 11q23. Conversely, alterations observed in MDS are mostly unbalanced, such as -7/del(7q), -5/del(5q), +8, dup(1q), del(20q), del(11q), del(12p)/t(12p), del(17p)/iso(17q), del(18q), +21q, del(13q), and +der(1;7)(q10;p10) (33, 34). Complex karyotypes are commonly reported in MDS, unlike in primary AMLs. The spectrum of mutated genes in AML and MDS is broadly overlapping. However, in AML, mutations affecting genes encoding tyrosine kinase receptor (*FLT3* and *KIT*), genes involved in the RAS pathway, and genes like *CEBPA, NPM1* and *IDH1/IDH2* are over-represented compared to MDS. Conversely, mutations affecting genes involved in splicing (*e.g.*, *SF3B1, U2AF1, SRSF2)*, as well as epigenetic regulators (*e.g DNMT3A*, *TET2*), are over-represented in MDS (33). In MDS, mutations are mainly C to T base transitions occurring in CpG dinucleotides, suggesting age-related methylated cytosine deamination (35).

### Models of Clonal Evolution Accounting for MDS Development and AML Transformation

As MDS naturally progresses to AML, a major research challenge is to uncover the genetic events leading the MDS-SCs to transform into LSCs. Intriguingly, one team has revealed that the proportion of clonal cells in the bone marrow does not vary between MDS and secondary AML stages (36). Thus, the blast cell percentage in MDS does not reflect clonality.

Whole genome sequencing techniques have established that the transformation into AML is defined by the persistence of a founder clone containing a large number of somatic mutations (several hundreds), and the emergence or growth of at least one subclone, carrying new mutations (several dozens to hundreds) (36). In agreement, Makishima et al. demonstrated that mutational diversity (reflected by the Shannon index) was higher in secondary AMLs than HR-MDS, and secondary AMLs were enriched with mutations affecting *FLT3, NPM1, NRAS, PTPN11, WT1, IDH1* and *IDH2* genes (35). In addition, MDS patients harboring mutations in one of these genes displayed a faster AML transformation (and thus reduced overall survival). Emergence of mutations affecting these genes could be investigated upon the follow-up of patients with MDS, in order to provide them early treatment which could be based on therapies targeting these abnormalities. These mutations are highly represented in primary AMLs as well.

Thus, a linear model was first established to account for leukemic transformation: a pre-myelodysplastic stem cell evolves into MDS-SCs, which in turn evolves into LSCs (37). However, Chen et al. recently revealed that stem cell compartments had a higher subclonal diversity than blast cells, at the MDS and secondary AML stages (38). They hypothesized that the relative quiescence of malignant stem cells exposed them to the accumulation of genetic abnormalities while aging. Indeed, it had already been emphasized that the quiescence of physiological stem cells led to the reparation of genome damage with highly infidel cell cycle-independent mechanisms (39). Moreover, stem cells displayed high basal expression of pro-survival genes, reducing elimination of damaged cells by apoptosis (39).

**Figure 1:**
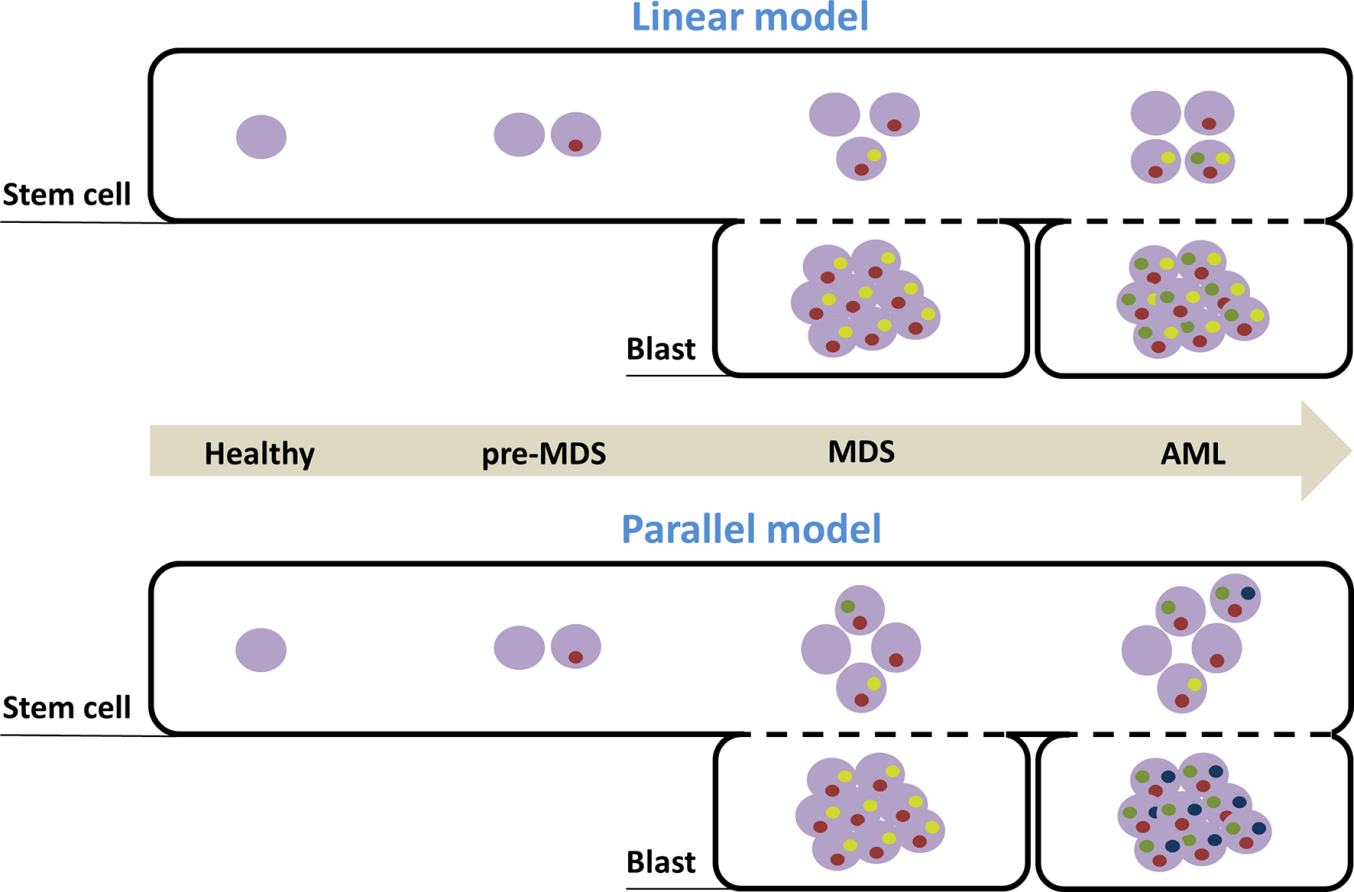
Models of clonal evolution accounting for MDS development and AML transformation. Distinct temporal stages of disease progression are represented with the arrow. Healthy cells are depicted by an empty purple circle. Inside purple circles, each colored circle (yellow, red, blue and green) symbolizes the presence of a distinct mutation. Combination of mutations defines specific clones. According to the linear model, clonal evolution in the stem cell compartment is simply sequential and cumulative and the dominant clone in MDS stage (found in the blast compartment) initiates AML, after acquisition of new mutations. According to the second model, pre-MDS-SCs acquire distinct additional mutations in parallel, that drive either MDS blast production or AML transformation. MDS, myelodysplastic syndrome; AML, acute myeloid leukemia; SC, Stem cells.

Single-cell sequencing has suggested that clonal evolution in the stem cell compartment was non-linear during MDS initiation and progression to AML, generating both a dominant clone as well as sub-clones; then, a reduced number of clones could be detected at the blast level (38). Thus, for the majority of patients, some subclones observed within AML blast cells were undetectable in blast cells at the MDS stage but were present in MDS-SCs (**Figure 1**).

LSCs display a phenotypic profile characteristic of progenitors in both primary and secondary AML (13). Thus, secondary AMLs could result from the successive acquisition of mutations, first in the HSC compartment and then in the progenitor compartment. Indeed, at least one genetic alteration carried by AML blasts could not be traced back to the Lin− CD34+ CD38− fraction in some patients (38).

## Part 3: Metabolic Characteristics of Malignant Stem Cells

LSCs are generally quiescent (mostly in G0 phase) (40, 41) and display low levels of reactive oxygen species (low-ROS) (42). Low-ROS LSCs are deficient in their ability to employ glycolysis, and preferably rely on oxydative phosphorylation. BCL-2 is upregulated in low-ROS LSCs and mediates oxidative respiration, driving them sensitive to BCL-2-targeting therapy (42). Moreover, LSCs aberrantly rely on exogenous cysteine intake to drive oxidative phosphorylation, cysteine depletion impairing the activity of electron transport chain and leading them to death (43). Targeting mitochondrial translation has demonstrated selective activity against LSC cells as well (44).

Ribosomal function effectors are overexpressed in AML LSCs compared to normal HSCs (45). The protein synthesis of physiological HSCs is extremely reduced compared to other hematopoietic cells and dysregulation of this synthesis hinders their function (46). In a murine model, Pten deficiency promoted leukemia partly by increasing protein synthesis (46).

Like LSCs, MDS-SCs are quiescent (mostly in G0 phase) (21). A recent study has demonstrated that MDS-SCs exhibit increased protein machinery activation and oxidative phosphorylation, as well as an increased use of the citric acid cycle in patients with HR-MDS, compared to healthy HSCs (21). Overexpression of ribosomal proteins is associated with a higher risk of transformation for HR-MDS patients (47). Targeting the protein synthesis (by omacetaxine), combined with targeting of oxidative phosphorylation (by venetoclax), has proven to be effective against MDS-SCs in immunocompromised mice engrafted with HR-MDS bone marrow cells (21). As this combination demonstrated low toxicity on healthy hematopoietic cells, a Phase 2A study was conducted to evaluate efficacy of omacetaxine mepesuccinate in HR-MDS population, yielding promising results (48). Surprisingly, MDS with isolated del(5q) have been associated with underexpression of genes involved in ribosomal biogenesis and translational control, and may be considered as ribosomopathies (47, 49). Moreover, mice hemizygous for *Rps6*, a gene encoding a ribosomal protein, displayed a phenotype very similar to MDS with isolated del(5q), dependent on P53 activation (50). In addition, patients diagnosed with other forms of LR-MDS displayed an underexpression of ribosomal proteins in their malignant stem cells (50).

## Conclusion

Functional experiments *in vitro* and *in vivo* have been used to define the phenotype of LSCs and MDS-SCs. MDS-SCs share a common phenotype with healthy HSCc, whereas LSCs phenotype is closer to that of healthy progenitors, mostly LMPP. The study of their gene expression profiles demonstrated that MDS-SCs are more likely to be derived from HSCs, while LSCs may rise from progenitors re-expressing stem cell transcriptional programs. The genetic abnormalities underlying MDS and AML are distinct, although they overlap to a large extent. Whole genome sequencing techniques have been able to establish that the progression of MDS to AML is defined by the persistence of a founder clone, and the emergence or growth of at least one subclone, carrying new mutations (mostly *FLT3, NPM1, NRAS, PTPN11, WT1, IDH1* and *IDH2)*. Single-cell genotyping has suggested that clonal evolution in the stem cell compartment is non-linear during MDS initiation and AML progression, generating both dominant clone and subclones, with a reduced number of clones detectable in the blast compartment. Malignant stem cells have demonstrated activation of the protein machinery and dependence on oxidative phosphorylation in both AML and HR-MDS. Targeting these metabolic abnormalities could prevent HR-MDS from progressing to AML.

## Author Contributions

RJ and TB designed the minireview and wrote the paper. All authors contributed to the article and approved the submitted version.

## Conflict of Interest

The authors declare that the research was conducted in the absence of any commercial or financial relationships that could be construed as a potential conflict of interest.

## Publisher’s Note

All claims expressed in this article are solely those of the authors and do not necessarily represent those of their affiliated organizations, or those of the publisher, the editors and the reviewers. Any product that may be evaluated in this article, or claim that may be made by its manufacturer, is not guaranteed or endorsed by the publisher.
